# Supplementary material for: Transition to siblinghood causes a substantial and long-lasting increase in urinary cortisol levels in wild bonobos
Source: eLife. 2022 Aug 30;11:e77227. doi: 10.7554/eLife.77227 (PMC9489214; doi:10.7554/eLife.77227)
Supplement: Supplementary file 1. — Green: classic interaction term derived from a separate model calculation (see ‘Methods’). ID = individual; S-birth = sibling birth; * before = before sibling birth; * early after = 7 and 4.5 months following sibling birth for cortisol and neopterin, respectively; * late after = time following early after. Data points are physiological measures corrected for specific gravity (SG). All smooths are not controlled for age to show cumulative pattern. [file elife-77227-supp1.docx]

Supplementary File 1: General additive mixed model results for physiological changes (urinary cortisol and urinary neopterin levels; all log-transformed) in the older offspring seven years before and after sibling birth. Green: Classic interaction term derived from a separate model calculation (see methods section). ID: Individual. S-birth = sibling birth, * before = before sibling birth, * early after = 7- and 4.5-months following sibling birth for cortisol and neopterin, respectively, * late after = time following early after. Data points are physiological measures corrected for specific gravity (SG). All smooths are not controlled for age to show cumulative pattern.
