# Supplementary material for: Transition to siblinghood causes a substantial and long-lasting increase in urinary cortisol levels in wild bonobos
Source: eLife. 2022 Aug 30;11:e77227. doi: 10.7554/eLife.77227 (PMC9489214; doi:10.7554/eLife.77227)
Supplement: Supplementary file 2. — sib birth = sibling birth; total T3 = total triiodothyronine. [file elife-77227-supp2.docx]

Supplementary File 2: Number of individuals and samples / data points (in brackets) for each physiological marker or behavior shown for each sex in relation to sibling birth. Sibling birth = sib birth, total triiodothyronine = total T3

|  |  | Cortisol |  | Neotperin |  | Total T3 |  |
| --- | --- | --- | --- | --- | --- | --- | --- |
| Time to sib birth | | Female | Male | Female | Male | Female | Male |
| before | years |  |  |  |  |  |  |
|  | >5 | 2 (3) | 1 (6) | 2 (3) | 1 (6) | 2 (3) | 1 (6) |
|  | 5>3 | 8 (23) | 3 (12) | 8 (22) | 3 (12) | 8 (23) | 3 (12) |
|  | 3>1 | 14 (49) | 5 (18) | 14 (47) | 5 (18) | 14 (49) | 5 (18) |
|  | 1 till birth | 13 (27) | 6 (13) | 13 (26) | 6 (13) | 13 (27) | 6 (13) |
| after |  |  |  |  |  |  |  |
|  | birth<1 | 14 (35) | 6 (15) | 14 (35) | 6 (15) | 14 (35) | 6 (15) |
|  | 1>3 | 11 (51) | 6 (15) | 11 (51) | 6 (15) | 11 (51) | 6 (15) |
|  | 3>5 | 7 (24) | 2 (12) | 7 (23) | 2 (12) | 7 (24) | 2 (12) |
|  | 5>7 | 4 (8) | 2 (8) | 4 (8) | 2 (8) | 4 (8) | 2 (8) |
|  |  |  |  |  |  |  |  |
|  |  | Behaviour datapoint | |  |  |  |  |
| Time to sib birth | | Female | Male |  |  |  |  |
| before | years |  |  |  |  |  |  |
|  | >2 | 5 (60) | 4 (17) |  |  |  |  |
|  | 2>1 | 6 (94) | 5 (82) |  |  |  |  |
|  | 1>birth | 8 (118) | 5 (105) |  |  |  |  |
| after |  |  |  |  |  |  |  |
|  | birth<1 | 7 (75) | 5 (40) |  |  |  |  |
|  | 1>2 | 2 (31) | - |  |  |  |  |
|  | >2 | 4 (73) | 1 (9) |  |  |  |  |
|  |  |  |  |  |  |  |  |
|  |  |  |  |  |  |  |  |
